# Risk factors for *Toxoplasma gondii* infection in dogs: a serological survey

**DOI:** 10.1186/s13028-024-00734-0

**Published:** 2024-03-25

**Authors:** Filippo Maria Dini, Laura Stancampiano, Giovanni Poglayen, Roberta Galuppi

**Affiliations:** Department of Veterinary Medical Sciences, Alma Mater Studiorum - University of Bologna, Bologna, Italy

**Keywords:** Dog, Risk factors, Serology, *Toxoplasma Gondii*

## Abstract

**Background:**

Dogs, as well as a wide variety of other warm-blooded animals, act as intermediate host of *Toxoplasma gondii*. In dogs, most cases of toxoplasmosis are subclinical, although clinical disease has been sporadically reported. Beyond its role in diagnostic pathways, seropositivity also functions as a reflection of the parasite’s spread within the dog’s living environment. The aim of the present study was to evaluate the possible risk factor associated with seropositivity to *T. gondii* in dogs in Central-Northern Italy, analysing 120 dogs sera for the presence of IgG antibodies by indirect fluorescence antibody test (IFAT).

**Results:**

The population examined was composed of 54.2% hunting dogs, 24.2% companion dogs, 14.2% truffle dogs and 7.5% watchdogs. Thirty-four (29.2%) dogs tested positive for *T. gondii* IgG, with titres ranging from 1:40 to 1:1280. Seroprevalence and antibodies titres were not related to dog gender, age or function. The logistic regression and ordered logistic regression results indicated that seroprevalence, and antibody titres were significantly higher in dogs cohabiting with cats, exhibiting coprophagy habits, and living constantly outdoors. Notably, the lifestyle factor showed the highest odds-ratios in the study: dogs living constantly outdoors were found to be at approximately 5 times greater risk of testing positive and having higher antibody titres compared to dogs living both indoors and outdoors.

**Conclusion:**

Both logistic and ordered logistic regression results support the key role of living with cats, engaging in coprophagy behaviours, and maintaining an outdoor lifestyle in increasing the risk of *T. gondii* infection in dogs. These identified risk factors collectively suggest that both ingesting oocysts, as observed through cat cohabitation and coprophagy, and engaging in predatory behaviours, as possible for outdoor living dogs, are indicating likely sources of *T. gondii* infection in this host species.

## Background

*Toxoplasma gondii* is a worldwide Apicomplexan protozoan that infects virtually all warm-blooded species including humans, livestock, birds, and pets [1]. It has been estimated that approximately one third of the world human population is infected with *T. gondii*, with the prevalence varying greatly depending on the geographical area [2, 3]. Domestic and wild felids are definitive host, harbouring the sexual stages of the parasite in their small intestine, releasing environmentally resistant oocysts. In all the other hosts, after the infection, asexual reproduction occurs, leading to bradyzoites cyst formation in several tissues. However, *T. gondii* can also undergo asexual reproduction in felids, that can therefore act also as intermediate host [4].

In many animal species, infection is typically subclinical, although toxoplasmosis can be lethal in several host species, including pets. *Toxoplasma* infection in dogs is often associated with low morbidity and mortality rates; indeed, primary clinical toxoplasmosis is infrequently observed in dogs, which is usually associated with previous immunosuppression [5]. The clinical aspects of canine toxoplasmosis range from nonspecific symptoms such as fever, lymphadenopathy, dyspnoea and gastrointestinal signs, to neurological signs characterized by epilepsy, cranial nerve deficits, tremors, ataxia, paresis, and paralysis. Other clinical features described are noise sensitivity, myositis, ocular diseases, and cutaneous signs associated with immunosuppressant therapies [5–10].

Seropositivity for *Toxoplasma* in dogs is not only an aid in the differential diagnosis of clinical cases, but has also epidemiological significance, reflecting the circulation of the parasite in the environment [1]. The seroprevalence of *T. gondii* in free-living animals, like stray dogs, serves as a valuable indicator for environmental contamination by *T. gondii* oocysts. These animals, sharing similar environmental risks with humans and wildlife, act as sentinel species. Monitoring their seroprevalence provides an indirect yet effective strategy to assess the distribution of *T. gondii* exposure in the environment [11–14]. The contact with oocysts may have other consequences besides infection of the dog. Indeed, it has been shown that dogs can act as mechanical transporters of *T. gondii* oocysts. They can excrete infective oocyst after ingestion of infected cat faeces, suggesting that coprophagy, with a subsequent intestinal passage by dogs, plays a role in the dissemination of *T. gondii* [15]. Additionally, dogs can vehicle oocysts on the fur after rolling over cat stool [16, 17]. As a result, mechanical transmission of *T. gondii* oocysts to humans can occur from dogs via their body surface, mouth, and feet [1].

*Toxoplasma gondii* infection has a cosmopolitan distribution, and seroprevalence in dogs depends on geographical region, living environment, and lifestyle of the dog. In general, according to the data reported in literature, the risk of infection with *T. gondii* increases throughout life, due to an increasing cumulative risk of exposure, and the seroprevalence is higher in rural than in urban areas [1, 18, 19]. In addition, it has been observed in several studies that dogs living outdoors have a higher risk of infection than indoor dogs [20–24].

As in humans, dogs can become infected with *T. gondii* through a variety of sources, including ingestion of water or food/feed containing sporulated oocysts, ingestion of raw or inadequately cooked meat containing cysts with bradyzoites, or transplacental infection [4]. Depending on the living environment of the dog, seropositivity may have different epidemiological implications. On the one hand, dogs living in anthropogenic areas have been shown to mirror seropositivity in humans, probably due to similar exposure to contaminated water and the environment [25]. On the other hand, stray or hunting dogs, whose *Toxoplasma* exposure is also related to the consumption of small wild prey, may be an indicator of the spread of the parasite in a wild area [20, 26].

The aim of the present work was to evaluate the risk factors for *T. gondii* infection in dogs with different uses, in an area of Italy where *Toxoplasma* infection previously have been detected in dogs [27] and in wild animals [27–29].

## Methods

**Fig. 1:**
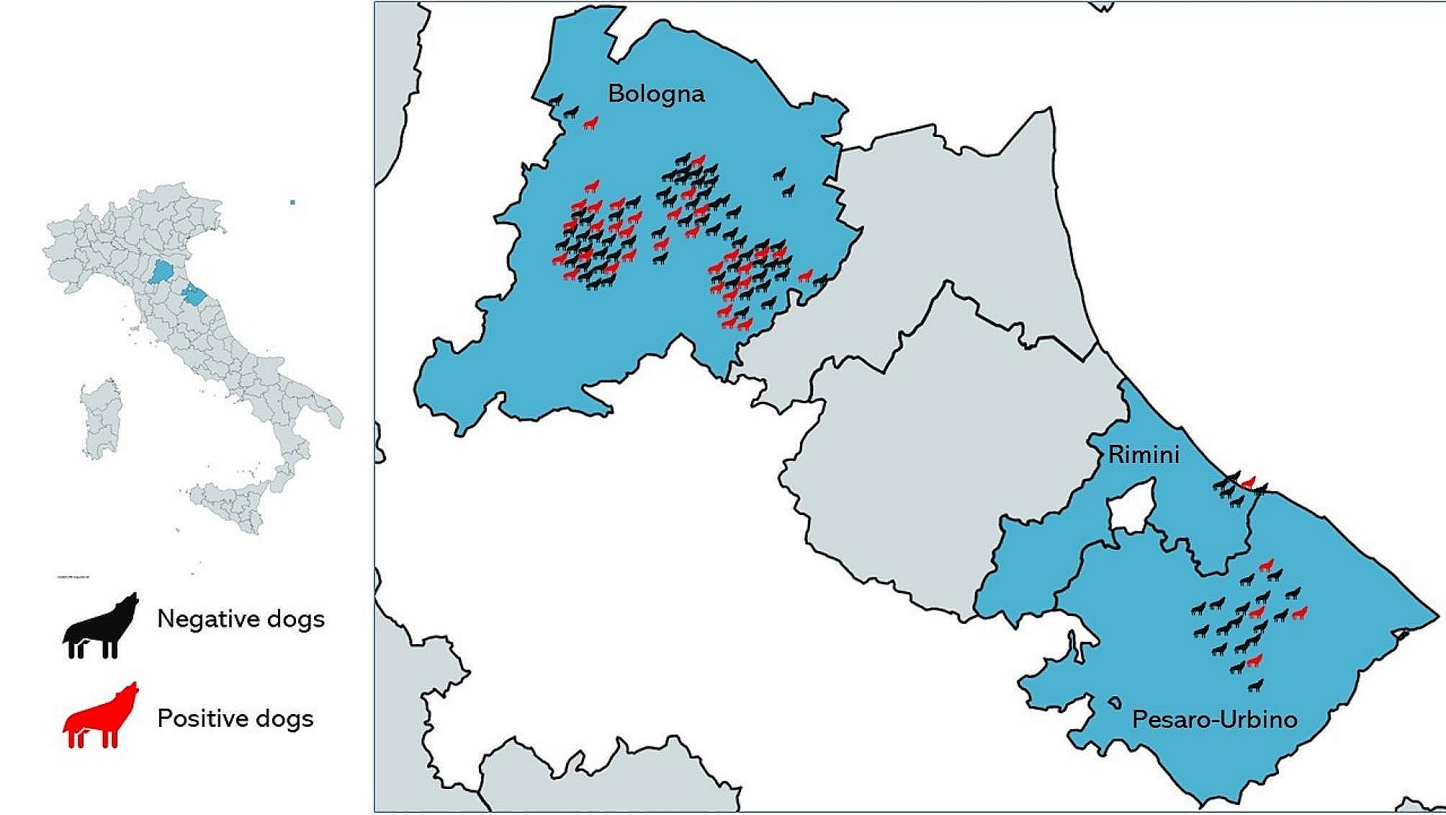
Geographical distribution of the dogs included in the study. Legend: Highlighted in blue are the provinces of Italy where sampling was conducted, specifically Bologna, Rimini, and Pesaro-Urbino. Black icons represent negative cases, while red icons denote positive cases *Toxoplasma gondii* infection, positioned within their respective geographic areas of origin

The study was based on convenience sampling involving the use of sera from 120 dogs collected for other research/diagnostic purposes from 20 municipalities in three provinces (Bologna, Rimini and Pesaro-Urbino) in 2018–2019 (Fig. 1). Blood sampling was carried out by venipuncture. Sera were obtained by centrifugation for 10 min at 980 g and stored at -20 °C until use. Inclusion criteria for enrolment included: regular outdoor access; no treatment for internal worms (including *Dirofilaria immitis* prophylaxis) in the month before the study, six months of age or older, and signed informed consent of the owner. A questionnaire was submitted to the owners in order to obtain information about age, gender, main use or function, housing (hosted or not in house during the night), lifestyle (living exclusively outdoor or hosted in house/boxes when not in activity), cohabitation with cats and coprophagy habits.

*Toxoplasma gondii* indirect fluorescent antibody test (IFAT) for IgG (MegaFLUO TOXOPLASMA g, MegaCor Diagnostik, Hoerbranz, Austria) was performed on serum samples, following the manufacturer’s instructions. Briefly, slides coated with *T. gondii* infected cells were probed with 20µL of serum diluted in phosphate-buffered saline (PBS) with a starting dilution of 1:40. Slides were incubated for 30 min at 37 °C and washed two times with PBS. Canine positive and negative control sera were included on each slide. The slides were thereafterprobed with 20µL of fluorescein isothiocyanate (FITC) conjugated anti-dog IgG antibody diluted in PBS at a concentration of 1:32 (Anti-Dog IgG-FITC antibody, Sigma-Aldrich, Saint Louis, MO) and incubated for 30 min at 37 °C. After two further washing steps with PBS, they were examined under a fluorescent microscope. The highest dilution showing fluorescence was the final antibody titre. Serum samples with antibody titre ≥ 1:40 were assessed positive, as 1:40 is the cut-off adopted for diagnostic purpose in different diagnostic facilities in the same area [27].

Statistical analysis was conducted using STATA 12.1. Prior to the analysis, the age of the dogs was grouped into three categories: ≤3 years, > 3 years and ≤ 7 years, > 7 years in order to obtain a uniform distribution of dogs in three age groups. The relationship between the prevalence of toxoplasmosis and various dog-related factors (such as age categories, gender, function, housing, lifestyle, cohabitation with cats, and coprophagy habits) was assessed using multivariable logistic regression. This approach allowed us to estimate the odds-ratio while keeping all other factors in the model constant; odds-ratio is a common approximation of the relative risk in cross-sectional surveys, indicating the likelihood of testing positive for toxoplasmosis in relation to each factor. To evaluate the relationship between antibody titres and the same dog-related factors considered for the prevalence analysis, multivariable ordered logistic regression was employed. Before this analysis, positive titres were log-transformed as log_2_(titre/10) to create a more manageable scale for calculations. The transformation did not alter the significance of the model, but it facilitated result interpretation. The dependent variable of the model, the transformed titre, represents an ordinal scale reflecting an underlying continuous measure, i.e., the concentration of antibodies. By using this model, we were able to estimate the odds ratio for each tested factor, considering the influence of each factor on increasing or decreasing antibody titres while keeping all other factors in the model constant.

## Results

The dogs were uniformly distributed in gender and age categories (Table 1). Concerning their function, hunting dogs made up the largest group (51.7%). All the dogs had regular outdoor access, as inclusion criteria, and most of them (71.7%) were not hosted in the house during the night. Nevertheless, only a small part of dogs lived exclusively outdoors (19.2%). The main function of the dogs influenced the housing and the lifestyle: all the hunting dogs (100%) were hosted outside (in kennel boxes) during the night, significantly differing from the other categories (Chi-square test: *p* < 0.01). In fact, most pet dogs (96.5%), and some truffle dogs (20%) and watchdogs (22.2%) were housed inside the owners’ homes during the night. On the other hand, considering lifestyle, hunting dogs lived in kennels when not actively engaged in hunting activities, but only 12.7% of them had a complete outdoor lifestyle (not differing significantly from pets: 3.4%). The predominant lifestyle for watchdogs was to remain outdoor (88.9%), differing from the other categories (Chi-square test: *p* < 0.05).

**Table 1:** Descriptive statistics and serological test results

	Category	n. dogs tested	Relative distribution %	n. positive at IFAT	Seroprevalence %	95% CI	
Gender	Male	67	58.8	22	32.8	21.1–44.6	
	Female	53	44.2	13	24.5	11.2–37.9	
Age groups	6 m– 3y	37	30.8	8	21.6	8.4–34.9	
	> 3–7 years	45	37.5	16	35.5	21.6–49.5	
	> 7 years	38	31.7	11	28.9	14.6–43.3	
Use	Pet dog	29	24.2	6	20.7	6.0-35.4	
	Watchdog	9	7.5	4	44.4	12.0-76.9	
	Hunting dog	62	51.7	19	30.6	19.2–42.1	
	Truffle dog	20	16.7	6	30.0	9.4–50.6	
Housing	House	34	28.3	8	23.5	9.3–37.7	
	Outside	86	71.7	27	31.4	21.6–41.2	
Lifestyle	Indoor/outdoor	97	80.8	22	22.7	14.4–31.0	
	Outdoor	23	19.2	13	56.5	36.0-76.5	
Cohabitation with cats	No	73	60.8	18	24.6	14.8–34.5	
	Yes	47	39.2	17	36.2	22.5–49.9	
Coprophagy	No	87	72.5	16	18.4	10.3–26.5	
	Yes	33	27.5	19	57.57	40.7–74.4	

Legend: CI = confidence interval.; IFAT = Indirect Fluorescent Antibody Test

Of the dogs in the study, 39.2% were reported to cohabit with cats, irrespective of the function of the dog. Interestingly, the habit of coprophagy, i.e., consuming faeces, was primarily observed in truffle dogs, with 65% of them exhibiting this behaviour. Companion dogs ranked second, with 50% of them having records of coprophagy according to the owner report.

Concerning the serological analysis, 35 out of 120 serum samples examined tested positive for *T. gondii* antibodies, resulting in a seroprevalence of 29.2% (95% CI = 21.1-37.2%).

The logistic regression results (Table 2) indicate that the seroprevalence, representing the probability of having been infected, is significantly higher in dogs cohabiting with cats, exhibiting coprophagy habits, and living constantly outdoors. This finding is consistent with the results from the ordered logistic regression (Table 3), where the antibody titres were significantly higher in dogs living with cats, having coprophagy habits, and constant outdoor living. Notably, the lifestyle factor showed the highest odds-ratios in the study. Dogs living outdoors constantly were found to be at approximately five times greater risk of testing positive and having higher antibody titres compared to dogs living both indoors and outdoors. In seropositive dogs, there appears to be a tendency for the antibody titres to increase with the age category; however, the differences observed were not statistically significant.

**Table 2:** Result of the logistic regression model having seropositivity as dependent variable

		O.R.	95% C.I.		*p*-value
Gender	Male	ref			
	Female	0.457	0.178–1.177		0.105
Age group	6 m– 3years	ref			
	> 3–7 years	1.975	0.650–6.003		0.230
	> 7 years	1.603	0.492–5.220		0.433
Use	Pet dog	ref			
	Watchdog	0.630	0.077–5.133		0.666
	Hunting dog	1.958	0.608–5.133		0.260
	Truffle dog	0.570	0.110–2.941		0.502
Lifestyle	Indoor/outdoor	ref			
	outdoor	5.289	1.319–21.209		0.019
Cohabitation with cats	No	ref			
	Yes	2.783	1.058–9.645		0.038
Coprophagy	No	ref			
	Yes	3.250	1.095–9.645		0.034

Legend: The term “ref” refers to the reference category of the covariates. O.R = odds-ratio; C.I.=confidence interval

**Table 3:** Result of Ordered logistic regression model having the log-transformed titre as dependent variable

Category		O.R.	95% C.I.	*p*-value
Gender	Male	ref		
	Female	0.430	0.173–1.070	0.070
Age group	6 m– 3years	ref		
	> 3–7 years	2.515	0.837–7.556	0.100
	> 7 years	1.960	0.616–6.240	0.255
Use	Pet dog	ref		
	Watchdog	0.629	0.092–4.308	0.637
	Hunting dog	1.976	0.605–6.454	0.259
	Truffle dog	0.486	0.010–2.368	0.372
Lifestyle	Inside/outside	ref		
	Outside	5.370	1.607–17.945	0.006
Cohabitation with cat	No	ref		
	Yes	3.068	1.224–7.694	0.017
Coprophagy	No	ref		
	Yes	4.051	1.443–11.370	0.008

Legend: The term “ref” refers to the reference category of the covariates. O.R = odds-ratio; C.I.=confidence interval

## Discussion

In the current investigation, a comprehensive spectrum of factors encompassing age categories, gender, function, housing arrangements, lifestyle, cohabitation with cats, and coprophagy habits was systematically scrutinized to discern and assess the risk factors intrinsically associated with *T. gondii* infection in dogs.

Recent data available on *T. gondii* seroprevalence in dogs in different countries of the world are quite divergent, even in the context of the same country: in Brazil it varies from 7.9 to 48.8% [30, 31], in China, *T. gondii* prevalence ranges from 4.4 to 40.3% [32, 33]. Of owned dogs in Bangkok, Thailand, 7.9% were found to be *T. gondii* positive in 2021 [24]. In the Americas, varying prevalence rates of *T. gondii* infection in dogs have been documented. Notably, studies have reported a prevalence of 16.8% in Colombia [34], and higher rates of 21% and 42.8% in the United States [1]. Particularly noteworthy are the elevated seroprevalence figures recorded in Mexican dogs, with rates of 59% [35] 61.7% [36] and 67.3% [14]. Regarding Europe, the prevalence reported in dogs (from Spain and Poland) was about 30% [20, 37], showing a similarity with our results. The seroprevalence observed in the present study, quantified at 29.2%, corresponds with seroprevalence data recently revealed in Northern Italy [27] pertaining to owned dogs, but with no concurrent risk factor analysis. It is noteworthy that the seroprevalence figures available in Italy are marked by notable variability. In the Campania Region, a survey involving a canine cohort of 398 hunting dogs unveiled a prevalence of 24% [38], in accordance with our findings. In contrast, findings presented by Macrì et al. [39] in Rome, encompassing both public kennel occupants and privately-owned dogs, disclosed a prevalence of 64%. The conspicuous divergence in these infection indexes is attributed, in part, to the utilization of disparate cut-off titres for seropositivity determination, being 1:50 and 1:20, respectively, for the aforementioned studies [38, 39]. This variance in cut-off titres unquestionably has had impact on the reported prevalence figures. The overarching challenge arising from these dissimilarities is the absence of standardized serological techniques and universally accepted initial cut-offs for diagnosing dog toxoplasmosis. Information available in scientific literature shows that the cut-off values employed for serological diagnosis of *T. gondii* in dogs using IFAT vary between 1:16 and 1:64 [1]. The absence of a standardized approach compromises the comparability of epidemiological data across studies, thereby precluding a comprehensive analysis of the actual epidemiological landscape prevalent within a given region.

The outcomes of the logistic regression analysis offer notable insights into the factors associated with *T. gondii* infection in the canine population under study.

Firstly, it is noteworthy that the seroprevalence exhibited a noticeable increase in dogs cohabiting with cats. This observation aligns with the findings of the ordered logistic regression analysis, where higher antibody titres were consistently observed in dogs sharing a living environment with cats. This correspondence across both regression analyses reinforces the notion that feline cohabitation serves as a significant predictor of heightened *T. gondii* infection risk. Following the excretion of the parasite in the faeces of infected felids, *T. gondii* oocysts have the potential to contaminate soil [40]. Given the restricted spectrum of definitive host species for *T. gondii*, limited exclusively to felids, the distribution of oocysts within the soil does not occur randomly. Instead, there is a discernible propensity for oocysts to aggregate in proximity to or within sites of cat defecation [41, 42]. These factors imply that living alongside cats increases the probability of being exposed to an environment contaminated with *Toxoplasma* oocysts, consequently increasing the potential for infection in the dogs that share the living space with felids.

Secondly, the coprophagy habits exhibited a similar pattern of association. Dogs displaying this behaviour showed an increased likelihood of seropositivity, as substantiated by their high antibody titres. The inclination to coprophagy, predominantly observed in this study among truffle dogs, followed by pet dogs, seemed to be less prevalent among hunting dogs based on the data analysis. However, the unique housing conditions associated with this dog category might lead to an underestimation of this variable, as these animals frequently remain out of the owner’s direct observation, potentially resulting in a lack of documentation for this behaviour. Coprophagy is a common behaviour among dogs. Dogs may consume their own faeces, faeces of other dogs and/or faeces of other species [43], including cats. Given that cats can shed millions of oocysts through their faeces during the course of sexual reproduction of *T. gondii* [4], the consistent habit of coprophagy, where dogs consume feline stool, places them at a significantly heightened risk of infection through oocysts.

The identification of heightened infection risk in dogs with behaviours such as cohabitating with cats or engaging in coprophagy emphasizes the environmental origin of these infections. This underscores the important role of dogs as sentinels, highlighting their importance in detecting and signalling environmental contamination with *T. gondii* oocysts [11–14].

Thirdly, the consistent outdoor residency of dogs emerged as a particularly prominent risk factor. It is noteworthy that this is true regardless of the dog’s function.

Actually, it might be expected that hunting dogs, that can more easily engage in predatory behaviour and are more likely exposed to game meat, would have been at higher risk of infection [44] Our results, thanks to multivariable analysis that evaluated different covariates avoiding possible confounding effects among them, did not support this assumption disentangling the importance of function and lifestyle as risk factors.

The consistency of the association between toxoplasmosis and “living outdoor” underscores the significance of the outdoor environment as a risk factor; it implies that the dog is subjected to prolonged exposure to potential sources of infection, including environmental oocysts, feline faeces and potentially infected small mammals or avian prey, independently to their function.

## Conclusions

In essence, both the logistic and the ordered logistic regression findings substantiated the pivotal role of cohabitation with cats, coprophagy behaviours, and perpetual outdoor habitation in amplifying the risk of *T. gondii* infection among dogs. This comprehensive understanding of the interplay between these factors and infection likelihood contributes to the broader comprehension of the epidemiological landscape and underscores the necessity for targeted preventive strategies, particularly for dogs exhibiting these risk-associated behaviours and conditions. Furthermore, the results of our study indicate that gender, age category, and function do not have a significant influence on toxoplasmosis seroprevalence. Instead, the findings suggest that habits play a more substantial role as risk factors for this zoonotic agent, compared to the individual’s function or receptivity.

## Data Availability

All data are included in this published article.
